# Immunogenic Potency of Formalin and Heat Inactivated *E. coli* O157:H7 in Mouse Model Administered by Different Routes

**Published:** 2020

**Authors:** Nasim Arshadi, Seyed Latif Mousavi, Jafar Amani, Shahram Nazarian

**Affiliations:** 1. Department of Biology, Faculty of Basic Sciences, Shahed University, Tehran, Iran; 2. Applied Microbiology Research Center, Systems Biology and Poisonings Institute, Baqiyatallah University of Medical Sciences, Tehran, Iran; 3. Department of Biology, Imam Hossein University, Tehran, Iran

**Keywords:** *Escherichia coli* O157:H7, Formaldehyde, Hot temperature, Immunization, Mice, Vaccines

## Abstract

**Background:**

Enterohemorrhagic *Escherichia coli (E. coli)* (EHEC) O157:H7 is a major foodborne pathogen causing severe disease in humans worldwide. Cattle are important reservoirs of *E. coli* O157:H7 and developing a specific immunity in animals would be invaluable. The administration of Whole Cell Vaccines (WCV) is a well-established method of vaccination against bacterial infections. Route of administration, inactivation and using suitable adjuvant have significant effects on the characteristics and efficacy of WCV.

**Methods:**

In the present study, an attempt was made to evaluate the immunogenic potency of heat and formalin inactivated cells administered orally and subcutaneously in mouse model by ELISA. Mice pretreated with streptomycin were used as a model to evaluate the efficacy of subcutaneous versus oral administration of the vaccine. Following immunization, mice were infected with *E. coli* O157:H7 and feces were monitored for shedding.

**Results:**

Both forms of inactivated cells induced immune response and hence protection against infectious diseases caused by *E. coli* O157:H7. However, formalin inactivated cells of *E. coli* O157:H7 showed superior antigenicity compared to heat inactivated cells. Subcutaneous immunization of mice with both heat and formalin inactivated *E. coli* O157:H7 induced significant specific levels of IgG antibodies but did not lead to significant antigen-specific IgA rise in feces, whereas oral immunization elicited significant levels of IgG antibodies with some animals developing antigen-specific IgA in feces.

**Conclusion:**

Inactivated *E. coli* O157:H7 is highly immunogenic and can induce protective immune responses via oral immunization.

## Introduction

Enterohemorrhagic *Escherichia coli* (*E. coli*) EHEC O157:H7 is a major food and water-borne pathogen which causes diarrhea, bloody diarrhea, hemorrhagic colitis and thrombotic thrombocytopenic purpura in humans, especially in young children and the elderly ^1^. Hemolytic Uremic Syndrome (HUS) is the most serious sequela of *E. coli* O157:H7 infection that occurs on average in 4% of infected humans ^2^. A number of factors have been identified to contribute in *E. coli* O157: H7 colonization of gastrointestinal epithelium, including fimbriae/pili, autotransporters, outer membrane proteins, flagella and Type III Secretion System (T3SS) ^3^. Intestinal colonization of pathogenic bacteria and release of Shiga toxins are important factors in infection of EHEC ^1^. Cattle are the primary animal reservoir of the gastrointestinal pathogen which can be directly acquired from beef/dairy products or indirectly *via* fecal shedding into the environment leading to contamination of other products or water supplies ^3^. Because of this, majority of EHEC control studies are focused on the eradication of this bacterium from the gastrointestinal tract of ruminants, whether by improved breeding practices or by vaccination ^4^.

Currently, there are few effective interventions to reduce the risk of this infection. Antibiotics are still effective treatment for O157 infection, while their usage promotes release of EHEC Shiga toxins, which increases the chance of complicating HUS ^5^.

The management of HUS requires control of bleeding, anemia, fluid and electrolyte imbalances, and other sequelae ^6^. Thus, vaccination remains one of the most promising pathways against *E. coli* O157:H7 infection. Reducing *E. coli* O157:H7 in the cattle could decrease the risk of infection in human. For this purpose, several vaccines have been developed in animal models which include recombinant proteins like Stx1/2, intimin, EspA, fusion proteins of A and B Stx subunits, a virulent ghost cells of EHEC O157:H7, live attenuated bacteria expressing recombinant proteins, recombinant fimbrial proteins and DNA vaccines ^6^.

The administration of Whole Cell Vaccines (WCV) is one of the well-established methods of vaccination against bacterial infections. The main advantages of WCV include the presentation of many antigens particularly the protective ones. Moreover, minimal chances of side effects when given non-parenterally, zero virulence potential, and adjuvant-like character can be enumerated as other favorable features. Inactivated vaccines have been prepared by a variety of methods. Formalin and heat inactivation are the most commonly utilized methods for WCV ^7^.

The aim of this study was to evaluate the efficacy of inactivated bacteria as a vaccine. Since in the WCV, antigens are provided in the natural form with known and unknown immunogens together, they produce a strong and enduring immune response. But recombinant subunit vaccines have some limitations, such as booster shots to get ongoing protection against diseases. Vaccination with formalin or heat inactivated bacteria administered orally or subcutaneously to block colonization of *E. coli* O157:H7 on small intestine has been compared.

## Materials and Methods

### Bacterial strains and culture conditions

Standard reference strains of *E. coli* O157:H7 ATCC: 35218 stored at −80°*C* in Luria-Bertani (LB) broth containing 20% glycerol, were grown on LB broth at 37°*C* with aeration of 150 *rpm* up to the late exponential phase.

### Strain characterization

The gene coding for rfbE was amplified from genomic DNA extracted from *E. coli* O l57:H7 for strain confirmation. Primers used for amplification of rfbE gene were gifted by Dr. S. Nazarian (Imam Hussein University, Tehran, Iran).

PCR reaction mixture contained 3 *mM* of MgCl_2_, 0.4 *mM* of each dNTP, 1×PCR buffer, 1 *U* of Taq DNA polymerase (Fermentas), 1 *μl* of DNA template and 0.4 *pm* of each primer. Temperature conditions included initial denaturation at 94°*C*/4 *min*, denaturation at 94°*C*/40 *s*, hybridization at 57°*C*/1 *min*, and polymerization at 72°*C*/1 *min* for 32 PCR cycles. The primers used for confirmation of bacterial strain are detailed in table 1.

**Table 1.** Primer sequences used in this experiment

**Primer**	**Sequence**	**PCR product (length; *bp*)**
**rfbE forward**	5-GTGCTTTTGATATTTTTCCGAGTAC-3	239
**rfbE reverse**	5-TTTATATCACGAAAACGTGAAATTG-3	239

### Preparation of formalin and heat inactivated bacteria

*E. coli* O157:H7 was cultured in LB at 37°*C* for 14 *hr*. For immunogenic studies, bacteria were grown to OD600: 0.6 and collected and washed thrice in sterile Phosphate-Buffered Saline (PBS, 137 *mM* NaCl, 2.7 *mM* KCl, and 4.3 *mM* Na_2_HPO_4_, pH=7.2) by centrifugation at 8000×*g* for 15 *min* at 4°*C*.

Cells were mixed with 0.4% formalin (MERCK, Germany) in PBS (pH=7.2) at 37°*C* for 1 *hr* in shaker, and then incubated for 18 *hr* at 4°*C*. Formalin-killed cells were collected at 10000×*g* for 30 *min* at 4°*C* and the cell pellet was washed with sterile PBS, resuspended in PBS to a final volume of 10^8^ and 10^9^ Colony Forming Units (CFU). The sterility test of formalin inactivated cells was performed onto Sorbitol Mac-Conkey agar (SMA) (Que lab, Canada), and incubation was done for 7 days at 37°*C* ^8–11^.

To prepare heat killed *E. coli* O157:H7, bacteria were suspended in PBS and centrifuged at 8,000 *g* for 15 *min*. The resulting pellet was washed thrice and resuspended in PBS with concentration of 10^8^ and 10^9^ CFU. Cell suspensions were heated at 70°*C* for 1 *hr* in normal pressure. In order to confirm complete killing, aliquots of the resulting cell suspensions were spread onto SMA plates and incubated at 37°*C* for one week ^12^.

### Animals

Female five-seven week-old BALB/c mice were obtained from Razi Institute (Karaj, Iran). Mice were housed in accordance with standard laboratory conditions with access to food and water ad libitum, in an environmentally controlled room with 12 *hr* light and dark cycles. A total of 30 mice were used to determine immunological response and rate of shedding post-challenge upon oral/subcutaneous administration of formalin and heat killed *E. coli O157*.

### Vaccination

Thirty female BALB/c mice weighing 20–25 *g* were randomly divided into six groups for two different experiments. In the first experiment, mice groups 1 and 2 subcutaneously received formalin and heat inactivated *E. coli* O157:H7, respectively. The third group was given PBS as a control. The immunization program is summarized in table 2.

**Table 2.** Immunized groups, route of administration and antigen quantitation

**Immunization groups**	**Route of administration**	**Antigen quantitation**
Inactivated *E. coli* O157:H7 by formalin and Alum	Subcutaneous	10^8^ bacteria
Inactivated *E. coli* O157:H7 by heat and Alum	Subcutaneous	10^8^ bacteria
PBS plus Alum	Subcutaneous	0.01 *M* PBS+equal volume Alum
Inactivated *E. coli* O157:H7 by formalin	Oral	10^9^ bacteria
Inactivated *E. coli* O157:H7 by heat	Oral	10^9^ bacteria
PBS	Oral	0.01 *M* PBS

Inactivated bacteria for subcutaneous immunization were suspended in PBS and emulsified with Alum as an adjuvant (Razi Institute, Karaj) with the ratio of 50:50 (volume/volume). The final amount of antigen for each subcutaneous immunization was equal to 10^8^ bacteria. Subcutaneous immunizations were done four times (1, 14, 28, 42 days). In the second experiment, 10^9^ bacteria were administered by oral gavage to mice 4 times (1, 7, 14, 21 days). Prior to oral immunization, 100 *μl* of sodium bicarbonate (10%) was administered by oral gavage to neutralize the acidic content of stomach ^9^. The immunization program is summarized in table 2.

### Serum collection

Blood samples were collected from all mice groups via retro-orbital plexus. The blood samples were incubated at 37°*C* for 1 *hr* and then incubated 1–2 *hr* at 4°*C*. Sera were collected by cold centrifugation for 15 *min* at 5000×*g*. The serum was collected and stored at −20°*C* until further use ^2,13^.

### Fecal immunoglobulin extraction

Seven days after the last oral vaccination, fresh fecal pellets were collected and added to the extraction buffer (0.01 *M* ice-cold PBS, pH=7.2, 0.5% Tween, and 0.05% sodium azide) at a ratio of 1 *ml* per 100 *mg* fecal pellets wet weight. Tubes were vortexed for 15 *min* at room temperature and the fecal suspensions were centrifuged at 3000×*g* for 15 *min* at 4°*C*. A portion of the supernatant (400 *μl*) was transferred to a sterile eppendorf tube containing100 *μl* glycerol to which 10 *μl* of Phenyl Methyl Sulphonyl Fluoride (PMSF, Sigma) solution was added and then vortexed briefly. Samples were centrifuged at 14,000×*g* for 15 *min* at 4°*C*. Supernatants were transferred to sterile tubes and stored at −20°*C* until use ^14^.

### Enzyme-linked immunosorbent assay (ELISA) for estimation of antibody titers

Analyses of serum IgG and IgA and mucosal IgA were carried out by indirect ELISA. Each well was added with 10^8^ appropriated bacteria in 100 *μl* of the coating buffer with pH=9.6 and incubated overnight at 4°*C* ^14,15^. The binding of residual protein was blocked with 100 *μl* of 1% BSA in PBST at 37°*C* for 1 *hr*. A series of negative controls were included concurrently with removing all ELISA components. Serially diluted serum samples (1:100 to 1:25, 600) and fecal pellet extracts (1:1 to 1:128) were added to wells and incubated 2 *hr* at 37°*C*. The wells were washed three times with PBST, and horseradish peroxidase goat anti-mouse IgG (Razi, 1:15000) or horseradish peroxidase goat anti-mouse IgA (sigma, 1:1200) was added to the wells. The plates were incubated at 4°*C* for 1.5 *hr*, then added with freshly prepared TMB substrate solution (Mono bind, USA) and incubated for 10 *min* at room temperature in the dark. The reaction was stopped by the addition of 3N H_2_SO_4_, and the absorbance was measured at 450 *nm* using a microplate reader (Sunrise remote, Tecan-Austria) to quantitate the amount of bound antibody. All samples were run in triplicate ^12,16–18^.

### Challenging the immunized mice

Mice were challenged 10 days after last immunization. Prior to challenges, mice were given drinking water containing streptomycin sulfate (5 *g/L*) to reduce the normal bacterial flora of gut. Following one day of treatment with streptomycin, mice were fasted overnight, and subsequently animals were inoculated by intragastric administration of approximately 10^10^ CFU of *E. coli* O157:H7. The extent of bacterial colonization was monitored every day for three weeks by quantitation of the *E. coli* O157:H7 shed into fecal pellets. *E. coli* O157:H7 fecal shedding was monitored by approximately 0.1 *g* of feces in 1 *ml* of LB broth followed by incubation at 37°*C* for 1–2 *hr* to allow the fecal pellets to soften. The mixture was then vortexed until the pellets were no longer visible. Serial dilutions of the supernatant were plated onto Sorbitol MacConkey agar plates containing tellurite. Plates were incubated overnight at 37°*C* and *E. coli* O157:H7colonies were enumerated. Bacterial colonies were tested for the O157 antigen by rfbE PCR ^2,17^.

### Statistical analysis

Data were analyzed using GraphPad Prism 6.01 (GraphPad Software Inc., California, USA). Before calculations, all values were log 10 transformed and mean value±SEM (Standard error of the mean) was calculated. Multigroup comparisons were performed using one way ANOVA. When the test was significant, further pairwise comparisons were performed using Tukey’s test. p-values of less than 0.05 were considered statistically significant.

## Results

### Strain characterization

**Figure 1.**
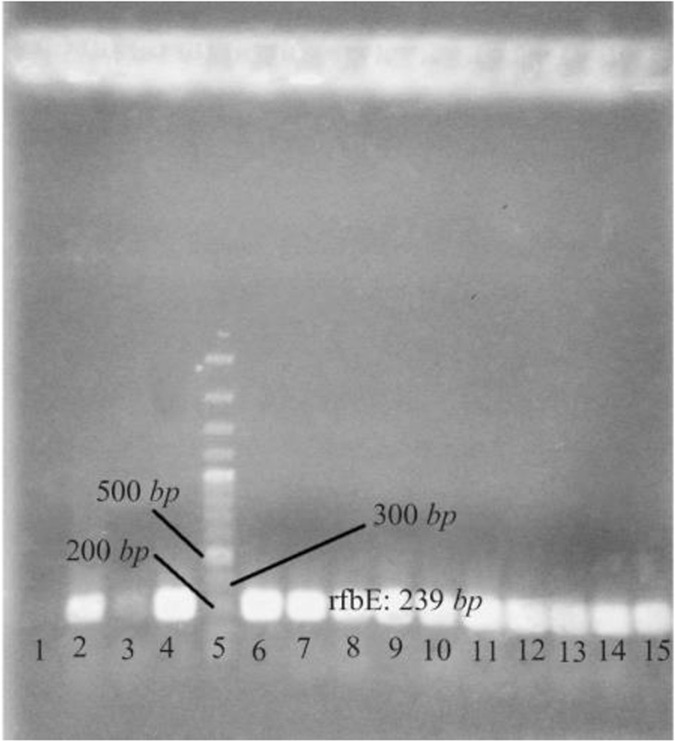
Agarose gel electrophoresis of PCR product of *E. coli* O157:H7 in order to confirm the bacteria. Lane 1: negative control; lane 2,4: positive control; lane 3: other bacteria; lane 5: 100 *bp* DNA ladder; lane 6–15: PCR product of the *rfbE* gene of *E. coli* O157:H7 grown in SMA.

The properties of strain used in this study were confirmed by PCR and biochemical tests. The rfbE gene of *E. coli* O157 encoding the O157 LPS is unique to the *E. coli* O157 serogroup (Figure 1) ^19^.

### Antibody responses to immunization

**Figure 2.**
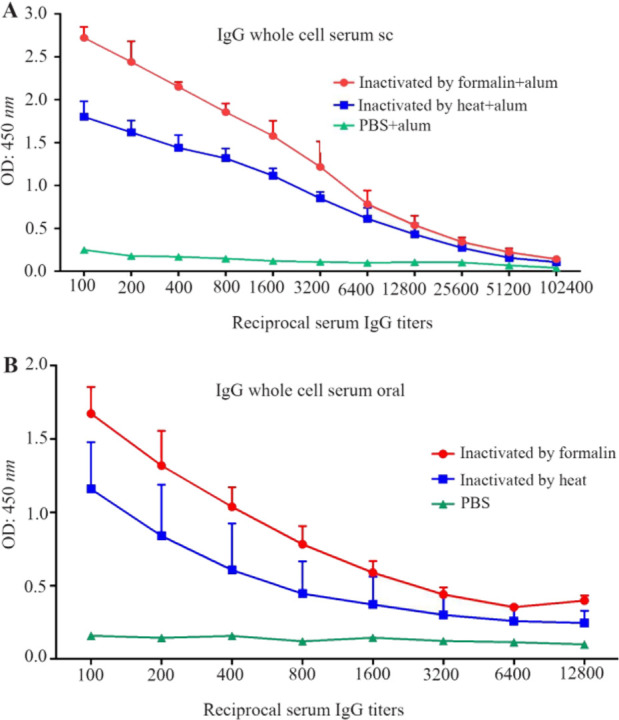
Specific serum IgG following oral versus subcutaneous immunization with inactivated bacteria. There were significant differences in antibody titer between immunized and control mice groups (p<0.05). A) IgG titer in the whole cell recipient group immunized subcutaneously, B) IgG titer in the whole cell recipient group immunized orally (Data represents the mean value±standard error of the three readings).

**Figure 3.**
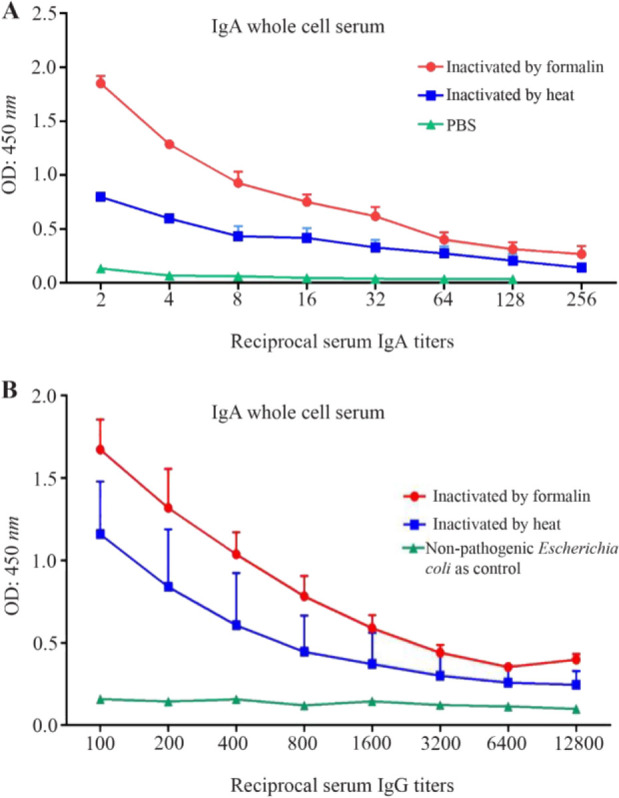
*E .coli* O157:H7 specific serum and fecal IgA following oral immunization (A) Serum IgA titer whole cell and (B) Fecal IgA titer whole cell in different mice groups 7 days after the final immunization (Data represents the mean value±standard error of the three readings).

Antibody titers of mice sera and fecal pellets after immunization with the inactivated *E. coli* O157:H7 were estimated. The mice were able to produce high serum IgG antibody titers after both subcutaneous and oral immunizations. Oral immunization resulted in significantly increased specific IgA titers (p<0.05) against *E. coli* O157:H7 in the feces and serum, compared to control mice. The serum IgG, IgA and fecal IgA ELISA results are summarized in figures 2 and 3.

### IgG antibody response in serum

Both oral and subcutaneous immunizations resulted in increased IgG antibody titers to *E. coli* O157:H7 compared to that of control mice (p<0.05) (Figure 2A and B).

### IgA antibody responses in serum and fecal extracts

IgA antibody responses to *E. coli* O157:H7 antigens in feces and serum were also studied. Oral immunization resulted in significantly increased specific IgA titers (p<0.05) against *E. coli* O157:H7 in the feces and serum compared to control mice (Figure 3A and B). Both the frequencies and magnitudes of IgA responses were lower than the IgG responses.

### Challenging of immunized mice with *E. coli* O157:H7

In order to determine whether *E. coli* O157:H7 antigens in immunized mice could reduce or prevent the *E. coli* O157:H7 shedding in feces, subcutaneously and orally immunized and non-immunized mice were orally infected with 10^10^ CFU of *E. coli* O157:H7.

**Figure 4.**
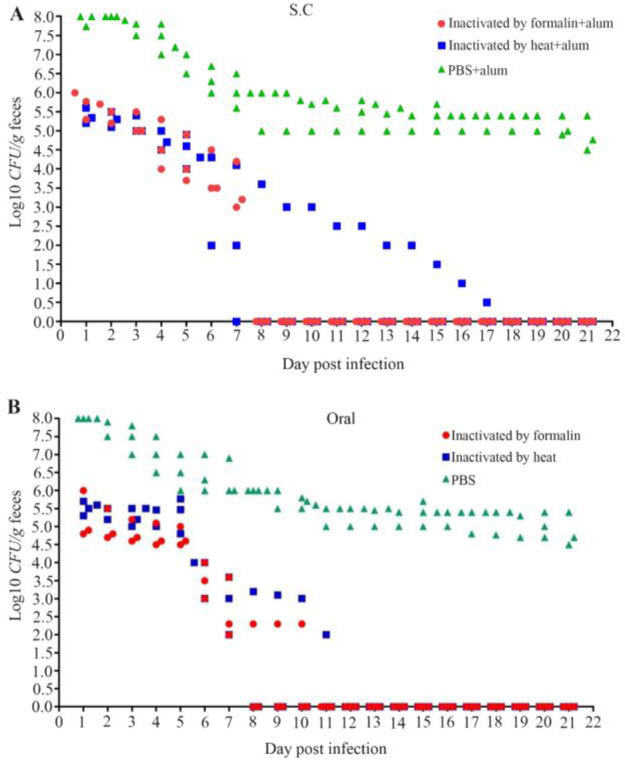
*E. coli* O157:H7 shedding in feces following subcutaneous (A) and oral (B) administration in mice. Immunized and nonimmunized mice were orally fed with 10^10^ *E. coli* O157:H7 and shedding was monitored in the feces for 21 days. Differences were considered significant whenever p<0.05. The limit of detection for plating was 10^10^ CFU/0.1 *g* feces. XY graphs are used for presenting data.

Shedding of the animals was monitored in feces. Shedding results are summarized in figures 4A and 4B. Non-immunized control mice shed high levels of *E. coli* O157:H7 (10^4^–10^8^ CFU) in their feces over the three weeks sampling period whereas shedding of nearly all immunized mice decreased gradually and stopped completely after 7–18 days (Figure 4). EHEC colonization in animals’ intestines was completely stopped after 8–10 days following immunization with formalin or heat inactivated bacteria through oral immunization route (some animals immunized with heat inactivated bacteria subcutaneously continued shedding till day 18^th^). Significant differences were observed in the colonization of immunized and non-immunized mice but no significant differences were observed between immunized mice (orally and subcutaneously) using Tukey’s multiple comparison test (p=0.050).

## Discussion

*E. coli* O157:H7 is the major pathogenic serotype of EHEC, a zoonotic enteric pathogen associated with sporadic outbreaks and illness ^17^. The prevalence of EHEC infections in humans has become a global public health problem ^16^, because exposure to antibiotics not only prevents pathogenesis but can even promote release of *E. coli* O157:H7 Shiga toxin by stimulation of the lytic cycle through bacterial SOS response ^20^. Therefore, controlling and eradicating *E. coli* O157:H7 infection is a challenging scientific problem and needs to be solved. Elimination of infectious agents, cutting off the transmission and protection of the vulnerable populations are three main elements in controlling communicable diseases. Vaccination is the most economic and effective means to reduce the incidence and protect humans and cattle against *E. coli* O157:H7 infection. Several vaccination approaches have been evaluated for EHEC. Among the vaccine candidates, those with immunological importance include Shiga toxins, virulence factors such as Tir, intimin, EspA and flagellin ^21^. These vaccines are mostly confined to few protein based antigens of T3SS and there might be other unknown antigens and excluding them reduces the efficacy of the vaccine. On the other hand, for many enteric pathogenic bacteria, due to the presence of polysaccharide on cell surface, protein based vaccines may not provide adequate protection. Specific polysaccharide of *E. coli* O157:H7 conjugated to endotoxin A of *Staphylococcus aeruginosa* (*S. aeruginosa*) was the first conjugate vaccine candidate ^22^. Although 6 months after the first injection, the antibody titers for IgG-LPS was 20 folds higher compared to pre injection with no significant adverse reaction, conjugate vaccines are chemically complex and their design, manufacturing and characterization is cumbersome. WCV can provide all potentially known and unknown antigens and overcome aforementioned problems and thereby offer an economically and potentially safe, effective means of preventing disease.

Differences in vaccine strains, conditions of growth, methods of inactivation, number of bacteria per dose, animal model and dosing schedules may explain the differences in efficacy of WCV ^23^. In this study, the effects of 4 different vaccine formulations were evaluated with 2 control groups. Different combinations of formalin/heat inactivated whole cells of *E. coli* O157: H7 were administered subcutaneously or orally. All vaccine formulations resulted in a significant reduction in total bacterial shedding (Figure 4).

The inactivation method can have significant effects on the characteristics of the vaccine ^7^. Both the heat and formalin inactivated vaccine formulations are shown to have complete protective capacity ^24^.

However, formalin inactivation of *E. coli* O157:H7 antigenicity showed better response compared with heat inactivated cells administered orally. This is due to the fact that surface antigens could retain their conformations on the formalin inactivated bacteria, but not on heat inactivated one. Animals immunized subcutaneously with heat killed *E. coli* O157:H7 containing 10^8^ CFU showed significant increase of IgG antibody titers in comparison with the control group ^5^. *Vibrio cholera* (*V. cholera)* inactivated with phenol and heat was found to be a comparatively poor immunogen. The replacement of phenol with formalin greatly increased the antibody titers. The antibody produced against the formalin-inactivation was capable of recognizing surface antigenic determinants of heterologous strains of *V. cholera.* Antibodies produced against pathogenic bacteria cannot identify normal flora bacteria, because the antigenic structure of normal flora bacteria and their attaching factors differ from pathogenic bacteria. In this vaccination, antibodies are being developed only against the attaching factors of pathogens ^23,25^. Heat Killed Multi-serotype Shigella (HKMS) immunogens were evaluated in a guinea pig and broad spectrum protection in a guinea pig against shigellosis was determined ^12^. Formalin-killed whole-cell preparation of five main diarrheagenic *E. coli* pathotypes, formulated as a combined vaccine candidate, offered protection against the five main diarrheagenic *E. coli* pathotypes in a single vaccine using mouse model ^9^.

Aside from the choice of immunogen, the route of administration is also important for developing a successful vaccine. In our study, vaccines were administered orally and subcutaneously.

For subcutaneous immunization, only high serum IgG antibody titers were obtained, whereas oral immunization resulted in not only high titers of serum IgG antibody but also high titers of IgA antibody in mice serum and feces (Figure 3A and B). Fan *et al* evaluated the efficacy of subcutaneous versus intranasal administration of the recombinant Tir as vaccine. Only high serum IgG antibody titers were raised with subcutaneous immunization, whereas intranasal immunization resulted in both high titers of serum IgG antibody as well as IgA antibody in mice serum and feces. Mucosal immunization stimulates immune responses and produces sIgA prior to bacterial colonization ^16^. Subcutaneous immunization of mice with type III secreted proteins induced significant EspA and Tir specific serum IgG antibodies but did not significantly induce any antigen-specific IgA in feces, whereas intranasal immunization elicited significant EspA and Tir specific serum IgG antibodies with some animals developing antigen-specific IgA in feces ^26^. Safe and tolerable oral WCV may be the most effective and practical way of preventing enteric disease and other mucosal diseases as well ^7^.

Shedding below 10^4^ *CFU/g* feces did not occur in the control group, indicating that vaccination may have a significant impact on *E. coli* O157:H7 carriage in mice through reducing mice-to-mice transmissions, even though vaccination may have little impact on the rate of clearance of bacterial infection.

In summary, the inactivated bacteria *E. coli* O157: H7 is highly immunogenic and can induce protective immune responses *via* oral immunization.

## Conclusion

WCV should continue to receive more attention as potentially effective, safe, and economical prophylactic and therapeutic treatments for infectious disease. Safe and tolerable oral WCV may be the most effective and practical way of preventing enteric and other mucosal diseases as well. The inactivation procedure may directly affect the functionality of the WCV, and selection of the inactivation method for WCV preparation should receive significant consideration.

## References

[1] Cai K Tu W Liu Y Li T Wang H Novel fusion antigen displayed-bacterial ghosts vaccine candidate against infection of Escherichia coli O157: H7. Sci Rep 2015;5: 17479. 26626573 10.1038/srep17479 PMC4667225

[2] Mohawk KL Melton-Celsa AR Zangari T Carroll EE O’ Brien AD Pathogenesis of Escherichia coli O157: H7 strain 86-24 following oral infection of BALB/c mice with an intact commensal flora. Microb Pathog 2010;48 (3–4):131–142. 20096770 10.1016/j.micpath.2010.01.003 PMC2834854

[3] McNeilly TN Mitchell MC Rosser T McAteer S Low JC Smith DG Immunization of cattle with a combination of purified intimin-531, EspA and Tir significantly reduces shedding of Escherichia coli O157: H7 following oral challenge. Vaccine 2010;28(5):1422–1428. 19903545 10.1016/j.vaccine.2009.10.076

[4] Garcia-Angulo VA Kalita A Torres AG Advances in the development of enterohemorrhagic Escherichia coli vaccines using murine models of infection. Vaccine 2013;31(32): 3229–3235. 23707170 10.1016/j.vaccine.2013.05.013 PMC3691335

[5] Yousif A Al-Taai N Mahmood N Humoral and cellular immune response induced by E. coli [O157: H7 and O157: H7: K99] vaccines in mice. Int J Immunol Res 2013;3(1):17.

[6] Saeedi P Yazdanparast M Behzadi E Salmanian AH Mousavi SL Nazarian S A review on strategies for decreasing E. coli O157: H7 risk in animals. Microb Pathog 2017; 103:186–195. 28062285 10.1016/j.micpath.2017.01.001

[7] Pace JL Rossi HA Esposito VM Frey SM Tucker KD Walker RI Inactivated whole-cell bacterial vaccines: current status and novel strategies. Vaccine 1998;16(16): 1563–1574. 9711805 10.1016/s0264-410x(98)00046-2

[8] Du Y Tang X Sheng X Xing J Zhan W Immune response of flounder (Paralichthys olivaceus) was associated with the concentration of inactivated Edwardsiella tarda and immersion time. Vet Immunol Immunopathol 2015;167(1–2):44–50. 26163937 10.1016/j.vetimm.2015.07.002

[9] Gohar A Abdeltawab NF Fahmy A Amin MA Development of safe, effective and immunogenic vaccine candidate for diarrheagenic Escherichia coli main pathotypes in a mouse model. BMC Res Notes 2016;9(1):80. 26860931 10.1186/s13104-016-1891-z PMC4748553

[10] Borde A Larsson A Holmgren J Nygren E Preparation and evaluation of a freeze-dried oral killed cholera vaccine formulation. Eur J Pharm Biopharm 2011;79(3): 508–518. 21757004 10.1016/j.ejpb.2011.06.009

[11] Jang YH Subramanian D Heo MS Efficacy of formalinkilled Pseudomonas anguilliseptica vaccine on immune gene expression and protection in farmed olive flounder, Paralich-thys olivaceus. Vaccine 2014;32(16): 1808–1813. 24530933 10.1016/j.vaccine.2014.01.088

[12] Nag D Sinha R Mitra S Barman S Takeda Y Shinoda S Heat killed multi-serotype Shigella immunogens induced humoral immunity and protection against heterologous challenge in rabbit model. Immunobiology 2015;220(11):1275–1283. 26210044 10.1016/j.imbio.2015.07.002

[13] Wan Cs Zhou Y Yu Y Zhao W Zheng XL B-cell epitope KT-12 of enterohemorrhagic Escherichia coli O157: H7: a novel peptide vaccine candidate. Microbiol Immunol 2011;55 (4):247–253. 21272063 10.1111/j.1348-0421.2011.00316.x

[14] Bao S Beagley KW Murray AM Caristo V Matthaei KI Young IG Intestinal IgA plasma cells of the B1 lineage are IL-5 dependent. Immunology 1998;94(2): 181–188. 9741339 10.1046/j.1365-2567.1998.00512.x PMC1364203

[15] Nygren E Holmgren J Attridge SR Murine antibody responses following systemic or mucosal immunization with viable or inactivated Vibrio cholerae. Vaccine 2008; 26(52): 6784–6790. 18951939 10.1016/j.vaccine.2008.10.011

[16] Fan HY Wang L Luo J Long BG Protection against Escherichia coli O157: H7 challenge by immunization of mice with purified Tir proteins. Mol Biol Reports 2012; 39(2):989–997. 10.1007/s11033-011-0824-0 21567195

[17] Zhang XH He KW Zhang SX Lu WC Zhao PD Luan XT Subcutaneous and intranasal immunization with Stx2B–Tir–Stx1B–Zot reduces colonization and shedding of Escherichia coli O157: H7 in mice. Vaccine 2011;29(22): 3923–3929. 21338683 10.1016/j.vaccine.2011.02.007

[18] Amani J Salmanian AH Rafati S Mousavi SL Immunogenic properties of chimeric protein from espA, eae and tir genes of Escherichia coli O157:H7. Vaccine 2010;28(42): 6923–6929. 20709010 10.1016/j.vaccine.2010.07.061

[19] Mousavi SL Rasooli I Nazarian S Amani J Simultaneous detection of Escherichia coli O157: H7, toxigenic Vibrio cholerae, and Salmonella typhimurium by multiplex PCR. Arch Clin Infect Dis 2009;4(2):97–103.

[20] Croxen MA Law RJ Scholz R Keeney KM Wlodarska M Finlay BB Recent advances in understanding enteric pathogenic Escherichia coli. Clin Microbiol Rev 2013;26(4):822–880. 24092857 10.1128/CMR.00022-13 PMC3811233

[21] Pacheco AR Sperandio V Shiga toxin in enterohemorrhagic E. coli: regulation and novel antivirulence strategies. Front Cell Infect Microbiol 2012;2:81. 22919672 10.3389/fcimb.2012.00081 PMC3417539

[22] Konadu EY Parke JC Jr Tran HT Bryla DA Robbins JB Szu SC Investigational vaccine for Escherichia coli O157: phase 1 study of O157 O-specific polysaccharide-Pseudomonas aeruginosa recombinant exoprotein A conjugates in adults. J Infect Dis 1998;177(2):383–387. 9466525 10.1086/514203

[23] Cryz S Fürer E Germanier R Effect of chemical and heat inactivation on the antigenicity and immunogenicity of Vibrio cholerae. Infect Immun 1982;38(1):21–26. 7141690 10.1128/iai.38.1.21-26.1982 PMC347690

[24] Mwirigi M Nkando I Aye R Soi R Ochanda H Berberov E Experimental evaluation of inactivated and live attenuated vaccines against Mycoplasma mycoides subsp. mycoides. Vet Immunol Immunopathol 2016;169: 63–67. 26827840 10.1016/j.vetimm.2015.12.006

[25] Nazarian S Gargari SL Rasooli I Alerasol M Bagheri S Alipoor SD Prevalent phenotypic and genotypic profile of enterotoxigenic Escherichia coli among Iranian children. Japanese J Infect Dis 2014;67(2):78–85. 10.7883/yoken.67.78 24647248

[26] Babiuk S Asper DJ Rogan D Mutwiri GK Potter AA Subcutaneous and intranasal immunization with type III secreted proteins can prevent colonization and shedding of Escherichia coli O157: H7 in mice. Microb Pathogenesis 2008;45(1):7–11. 10.1016/j.micpath.2008.01.005 18487034

